# Inhibiting DNA methylation alleviates cigarette smoke extract-induced dysregulation of Bcl-2 and endothelial apoptosis

**DOI:** 10.18332/tid/119163

**Published:** 2020-06-03

**Authors:** Huihui Zeng, Xianglong Kong, Hongliang Zhang, Yan Chen, Shan Cai, Hong Luo, Ping Chen

**Affiliations:** 1 Department of Respiratory Medicine, The Second Xiangya Hospital, Central South University, Changsha, China; 2 Research Unit of Respiratory Diseases, Central South University, Changsha, China; 3 Hunan Centre for Evidence-based Medicine, Changsha, China; 4 Department of Respiratory Medicine, The First Hospital of Changsha, Changsha, China; 5 Department of Emergency Medicine, The Second Xiangya Hospital, Central South University, Changsha, China

**Keywords:** cigarette smoke, endothelial apoptosis, Bcl-2, methylation

## Abstract

**INTRODUCTION:**

There is evidence that cigarette smoking participates in disease progression through endothelial apoptosis. Bcl-2 family proteins are essential and critical regulators of apoptosis. We explored whether Bcl-2 plays a role in cigarette smoke extract induced (CSE-induced) endothelial apoptosis. Furthermore, given the involvement of epigenetics in apoptosis and Bcl-2 expression, we hypothesized that CSE-induced apoptosis might be caused by gene methylation.

**METHODS:**

Human umbilical vascular endothelial cells (HUVECs) were treated with CSE, CSE plus 5-aza-2’-deoxycytidine (AZA, an inhibitor of DNA methylation), or AZA and phosphate-buffered saline (PBS). Endothelial apoptosis was determined by Annexin-V and propidium iodide staining. The expression levels of Bcl-2, Bax, and cytochrome C (cyt C) were assessed by immunoblotting and RT-PCR. The methylation status of the Bcl-2 promoter was observed by bisulfite sequencing PCR (BSP).

**RESULTS:**

The apoptotic index of endothelial cells in the CSE-treated group increased. Decreased expression of Bcl-2 and high methylation of the Bcl-2 promoter were observed after CSE treatment. AZA alleviated the endothelial apoptosis caused by CSE. AZA treatment also increased Bcl-2 expression along with decreased Bcl-2 promoter methylation.

**CONCLUSIONS:**

Inhibiting DNA methylation alleviates CSE-induced endothelial apoptosis and Bcl-2 promoter methylation. Bcl-2 promoter methylation might be involved in CES-induced endothelial apoptosis.

## INTRODUCTION

Cigarette smoking is a well-known risk factor for many diseases, such as chronic obstructive pulmonary disease, hypertension, and coronary heart disease, among others. Our previous research found that intraperitoneal injection of cigarette smoke extract (CSE) induced emphysema and injury of the cardiac system in mice^1^. There has been mounting evidence suggesting that cigarette smoking participates in disease progression through endothelial apoptosis^2,3^. It has long been established that cigarette smoke induces endothelial apoptosis^4,5^. However, the underlying mechanisms of the apoptosis process are still poorly understood. Apoptosis is a highly regulated program of cell death that can be regulated by Bcl-2 family proteins via mitochondrial maintenance^6,7^. These Bcl-2 family proteins consist of anti- and pro-apoptotic members. Interactions between the classic anti-apoptotic protein B-cell lymphoma-2 (Bcl-2) and the pro-apoptotic protein Bcl2–associated X protein (Bax) determine whether the mitochondria will release cytochrome c (cyt C), which is the initial factor of apoptosis^6^. Endothelial mitochondrial maintenance is highly susceptible to cigarette smoking-related damage, and the damage can persist after the cessation of smoking behavior^8^. This result suggests that there is an additional pathogenesis route beyond direct mitochondrial damage.

Some studies have indicated that methylation, an important epigenetic event, participates in the regulation of Bcl-2 and apoptosis^9,10^. Promoter methylation leads to the condensation of chromatin into a compact state, which is inaccessible to transcription factors, causing the downregulation of exon expression. A high methylation status of the Bcl-2 promoter results in reduced expression of Bcl-2 mRNA^10^. Recent studies have also demonstrated the involvement of epigenetics in smokers and ex-smokers^11^. A previous study from our group showed that hypermethylation of the Bcl-2 promoter took a part in CSE-induced emphysema^12^. Our team also demonstrated that inhibiting DNA methylation might protect endothelial progenitor cells from apoptosis^13^. Taken together, these data present a new possibility that inhibiting DNA methylation might recover the cigarette-induced aberrant methylation of the Bcl-2 promoter and prevent endothelial apoptosis.

Thus, the present study explored the effect of 5-aza-2′-deoxycytidine (AZA), inhibiting DNA methylation through DNA methyltransferase enzymes (DNMT), on Bcl-2 methylation status and endothelial apoptosis after treatment with cigarette smoke extract (CSE).

## METHODS

### Cell culture

Human umbilical vascular endothelial cells (HUVECs) were purchased from the American Type Cell Culture Collection (ATCC, lot number: CRL-1730) and were cultured in RPMI-1640 medium (GIBCO, Invitrogen Inc., Carlsbad, CA, USA) containing 10% heat-inactivated foetal bovine serum (GIBCO, Invitrogen Inc.) and 2 mM L-glutamine at 37°C in a humidified atmosphere with 5% CO_2_.

### CSE treatment of HUVECs

CSE was prepared as previously described^4,14^. Briefly, one unfiltered cigarette (China Tobacco Hunan Industrial CO, Ltd. Tar: 12 mg, Nicotine: 1.1 mg, Carbon Monoxide: 14 mg) was burned, and then, the smoke was passed through 25 mL of phosphate-buffered saline (PBS) using a vacuum pump. This 100% CSE was adjusted to pH 7.4, and particles and bacteria were removed by filter (Millex-GP syringe filter, Merck Millipore, DE). CSE was prepared fresh for each set of experiments. After our pilot study, this study chose the 5% CSE concentration to treat cells (Supplementary file). The 100% CSE was diluted in RPMI-1640 medium to obtain a 5% CSE medium.

After serum starvation for 24 hours, HUVECs were divided into two groups (CSE and control). The cells in the CSE group were supplemented with 5% CSE medium for 12 hours. The control group was supplemented with RPMI-1640 medium for 12 hours. During this exposure, the culture medium was replaced every 12 hours to prevent the depletion of essential nutrients. The cells were harvested for the determination of apoptosis and Bcl-2, Bax and cyt C expression levels.

### Inhibiting DNA methylation in cells

AZA (Sigma, St. Louis, MO, USA), inhibiting DNA methylation through DNMT, was diluted in RPMI-1640 medium to obtain 1 μM AZA medium. The AZA medium was adjusted to pH 7.4 and filtered through a 0.22 μm pore filter (Fisher, NH) to remove bacteria and large particles. The AZA medium was prepared fresh before each experiment.

After serum starvation, methylation-inhibited HUVECs were incubated in two groups (AZA+CSE, AZA). The AZA+CSE group was pre-treated with 1 μM AZA medium for 12 hours, followed by incubation with 5% CSE medium for next 12 hours. The AZA group was incubated with 1 μM AZA medium for next 12 hours, then followed by incubation with RPMI-1640 medium for 12 hours. During this exposure, the medium was replaced every 12 hours to prevent the depletion of essential nutrients. The cells were harvested for the determination of apoptosis and Bcl-2, Bax and cyt C expression levels.

### Apoptosis determination by Annexin-V and propidium iodide staining

Apoptosis was determined using an Annexin-V FITC apoptosis detection kit (Biosea Biotechnology Co., Ltd., Beijing, China) following the manufacturer’s instructions. Cells were harvested by centrifugation, resuspended in binding buffer, and successively incubated with 10 μL of Annexin-V FITC and 5 μL of propidium iodide (PI) stains at room temperature for 15 minutes while protected from light. Apoptosis levels were determined by flow cytometry following a FACSCalibur instrument (BD Biosciences, San Jose, CA, USA) and previous studies^15-17^. Cells that stained negative for PI and positive for Annexin-V were considered to be early apoptotic cells.

### Immunoblotting

HUVECs were homogenized immediately after they were harvested in a buffer containing RIPA lysis and PMSF (Solarbio, Beijing, China), according to the manual. The supernatant was separated after two cycles of centrifugation at 1000g for 10 minutes. The isolation of cytosolic fractions was performed following the instructions of the Mitochondria Isolation Kit for Cultured Cells (Pierce, USA). The concentration of both total and cytosolic protein was determined using a bicinchoninic acid (BCA) protein assay (Pierce, USA).

A total of 30 μg of protein were separated by SDS-PAGE (Beyotime, Beijing, China) and transferred to nitrocellulose (NC) membranes (Millipore, MA, USA). After the protein was transferred, the membranes were blocked with 5% non-fat milk (dry milk diluted in PBST, PBS containing 0.05% Tween) for 1 hour at room temperature. After they were blocked, the membranes were washed and incubated overnight with rabbit polyclonal antibodies at 4°C (anti-Bcl-2, Bax and cyt C; 1:1000 dilution; Cell Signaling Technology, USA). The membranes were washed four times with PBST and incubated with HRP-conjugated goat anti-rabbit IgG (Jackson Immuno Research Laboratories, USA) for 1 hour at room temperature. Bands were detected using an ECL kit (Thermo, USA), and the film was developed and fixed using a developer and fixer kit (Beyotime, Shanghai, China). The expression of each protein was detected and quantitated with Quantity-One software (Bio-Rad Laboratories, CA).

### RT-PCR

Total RNA was extracted from HUVECs as described by Rio et al.^18^. After extraction, the RNA was reverse-transcribed using the PrimeScript® RT reagent kit (Takara, Dalian, China) and assayed using SYBR® Premix Ex TaqTM following the manufacturer’s instructions. All of the primers were obtained from Sangon Shanghai, China (Table 1). Real-time polymerase chain reaction (PCR) was conducted on the Step-one ABI Real-time RT-PCR system. All mRNA expression levels were calculated relative to that of β-actin.

**Table 1:** Primers for RT-PCR

Bcl-2	forward	5’-CGCATCAGGAAGGCTAGAGTT-3’
	reverse	5’-CAGACATTCGGAGACCACACT-3’
Bax	forward	5’-AAGCTGAGCGAGTGTCTCAAG-3’
	reverse	5’-CAAAGTAGAAAAGGGCGACAAC-3’
cyt C	forward	5’-CCCCTGATACTCTTACACAGC-3’
	reverse	5’-AGTCTGCCCTTTCTTCCTTCTT-3’
β-actin	forward	5’-GCACCACACCTTCTACAATGAG-3’
	reverse	5’-GATAGCACAGCCTGGATAGCA-3’

### Analysis of Bcl-2 promoter methylation status

The Bcl-2 promoter was determined to exist between -3000 bp and +70 bp using the Transcriptional Regulator Element Database (Accession Number 19717, NM 000633). After searching for CpG islands in the UCSC Genome Browser, the CpG islands in the promoter (-213 bp to +70 bp) were detected and analyzed for their methylation status using bisulfite sequencing PCR (BSP). The primers (Table 2) for BSP were designed using MethPrimer (http://www.urogene.org/methprimer/) and were blasted and confirmed using methBLAST (http://medgen.ugent.be/methBLAST/). A genomic DNA extraction kit (Takara, Dalian, China) was used to extract DNA from cells. Bisulfite conversion of DNA was performed with EpiTect Bisulfate Kits (QIAGEN, Netherlands) following the manufacturer’s instructions. After bisulfite modification, nested PCR was performed. The premix solution for the first round contained bisulfite-modified DNA (2 μL), Taq (0.2 μL) (Takara, Dalian, China), a pair of primers (20 μm, 1 μL each), DEPC-treated H2O (11.8 μL; Takara, Dalian, China), and dNTPs (10 mM, 1 μL; Takara, Dalian, China). PCR cycling was performed as follows: denaturation at 94°C for 3 minutes; 30 cycles at 94°C for 30 seconds, at 50°C for 30 seconds, and at 72°C for 1 minute; followed by a final extension at 72°C for 5 minutes. For the second round, the primers were changed, and the same PCR cycling parameters were used. After amplification, the PCR products were cloned into the pMD-18T vector (Takara, Dalian, China) and sequenced. The methylation status was calculated by counting the number of methylated CpGs in all clones, and the methylation status was expressed as the percentage of total CpGs.

**Table 2:** Primers for BSP

Bcl-2	forward	5’-AGGAATTGGAATAAAAATTTTTTGTATT-3’
	reverse	5’-ACAACTTATAATAAATATACTTCATCACTA-3’

### Statistical analysis

A software package (SPSS 16.0; Statistical Product and Service Solutions, USA) was used to perform all statistical analyses. The values are described with means and standard deviation (SD). Student’s t-test, one-way ANOVA and Kruskal-Wallis tests were performed to evaluate each group of data and p-values less than 0.05 were considered statistically significant.

## RESULTS

### CSE induced apoptosis of HUVECs

**Figure 1:**
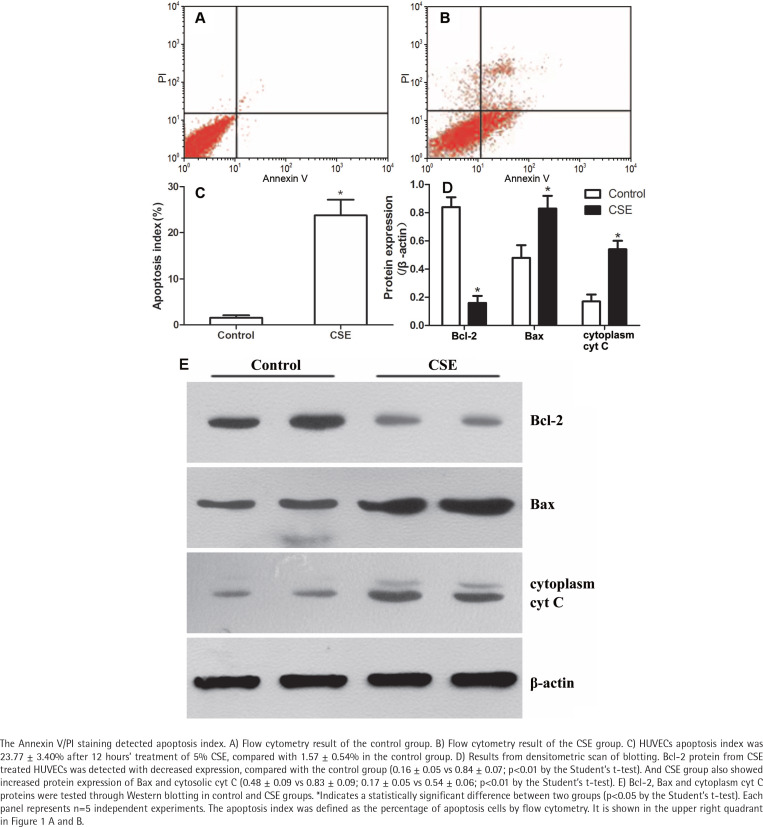
CSE induced apoptosis of HUVECs and regulated protein expression of Bcl-2, Bax and cytoplasmic cyt C

The Annexin V/PI staining showed that the apoptosis index of HUVECs was 23.77 ± 3.40% after CSE treatment, compared with 1.57 ± 0.54% in the control group (Figure 1 A, B, and C). As in our previous research^4,14^, the difference between the CSE and control groups was significant (p<0.05 by the Student’s t-test), supporting the idea that cigarette smoke induces endothelial apoptosis.

### CSE regulated Bcl-2, Bax and cytoplasmic cyt C expression in HUVECs

After a 12-hour incubation in CSE medium, HUVECs had lower Bcl-2 protein levels (0.16 ± 0.05 vs 0.84 ± 0.07; p<0.01 by the Student’s t-test, Figure 1 D and E). Since the balance of anti-apoptotic Bcl-2 and pro-apoptotic Bax determines the release of cyt C to initiate apoptosis, we measured the Bax and cytoplasmic cyt C levels in the CSE and control groups. Interestingly, Bax protein and cytoplasmic cyt C protein expression levels were abnormally elevated after CSE treatment (Figure 1 D and E). The ratio of Bcl2/Bax was decreased after CSE treatment, compared with the control group (0.19 ± 0.07 vs 0.78 ± 0.07; p<0.01 by the Student’s t-test).

**Figure 2:**
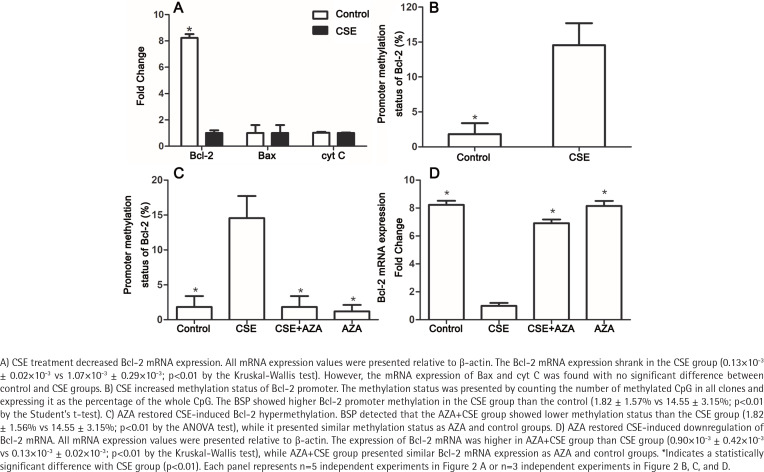
Inhibiting DNA methylation restored CSE-induced deregulation of Bcl-2 methylation and mRNA expression

To explore the mechanism by which Bcl-2 protein expression was reduced, the mRNA expression levels of Bcl-2, Bax and cyt C in the CSE and control groups were detected. As expected, the Bcl-2 mRNA expression was lower in the CSE group than in the control group (0.13×10^-3^ ± 0.02×10^-3^ vs 1.07×10^-3^ ± 0.29×10^-3^; p<0.01 by the Kruskal-Wallis test, Figure 2 A). However, the mRNA levels of Bax and cyt C showed no difference between the CSE and control groups (Figure 2 A), indicating that the regulation of Bax expression by CSE might be post-transcriptional.

### CSE increased the methylation status of the Bcl-2 promoter

It is widely accepted that gene transcription rates can regulate mRNA levels, and promoter methylation is an important mechanism of regulation to decrease transcription^19^. Previous studies have demonstrated that gene methylation, especially promoter methylation, participates in the regulation of Bcl-2 expression^20,21^. To investigate whether methylation participates in the Bcl-2 downregulation induced by CSE treatment, we measured methylation status in both the CSE and control groups. BSP showed higher Bcl-2 promoter methylation in the CSE group than in the control group (1.82 ± 1.57% vs 14.55 ± 3.15%; p<0.01 by the Student’s t-test, Figure 2 B). Therefore, Bcl-2 downregulation might be caused by a high degree of promoter methylation.

### Inhibiting DNA methylation restored the CSE-induced dysregulation of Bcl-2 methylation and expression

Due to the involvement of methylation in CSE-treated HUVECs, we hypothesized that inhibiting DNA methylation would reverse the downregulation induced by CSE. To test this hypothesis, we constructed control, CSE, AZA+CSE and AZA groups. AZA are types of inhibitors frequently used to investigate the methylation process. In this study, HUVECs were treated with AZA and CSE sequentially in the AZA+CSE group and were compared with HUVECs treated with CSE only, HUVECs treated with AZA only, and control HUVECs. BSP analysis showed that the AZA+CSE group was less methylated than the CSE group (p<0.01 by the ANOVA test, Figure 2 C), whereas it had a similar methylation level as the AZA and control groups (p>0.1, Figure 2 C).

**Figure 3:**
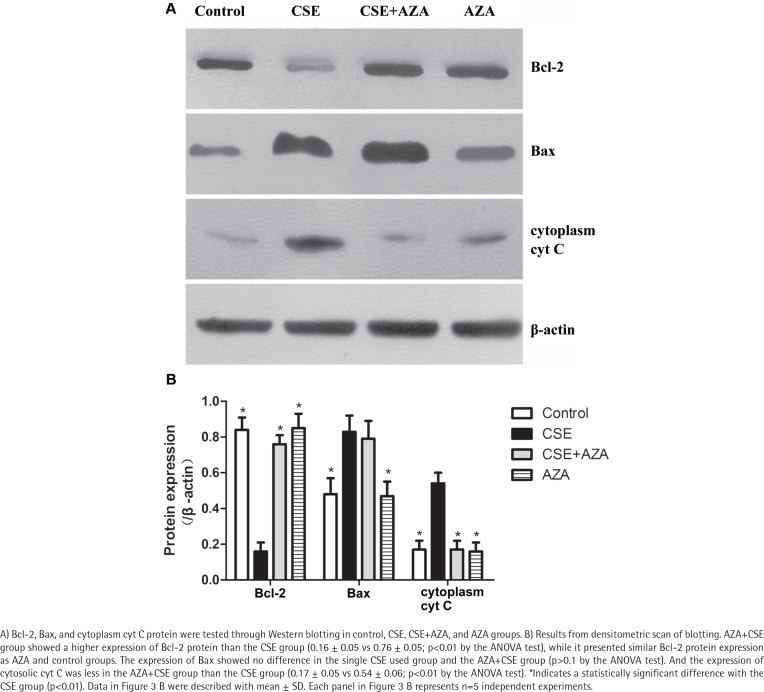
Inhibiting of DNA methylation restored CSE-induced downregulation of Bcl-2 protein expression

As expected, the expression of Bcl-2 mRNA was higher in the AZA+CSE group than in the CSE-only group (0.90×10^-3^ ± 0.42×10^-3^ vs 0.13×10^-3^ ± 0.02×10^-3^; p<0.01 by the Kruskal-Wallis test, Figure 2 D). An analysis of protein levels presented similar results: the AZA+CSE group showed a higher level of Bcl-2 protein expression than the CSE group (0.16 ± 0.05 vs 0.76 ± 0.05; p<0.01 by the ANOVA test, Figure 3). All of these results suggest that AZA pretreatment could restore Bcl-2 protein and mRNA expression.

Interestingly, immune blotting showed lower levels of cytosolic cyt C in the AZA+CSE group than in the CSE group (0.17 ± 0.05 vs 0.54 ± 0.06; p<0.01 by the ANOVA test, Figure 3). This finding indicates that the protein expression of cytosolic cyt C is also reduced by AZA pretreatment. On the other hand, the protein expression of Bax showed no difference in the CSE only and AZA+CSE groups (p>0.1 by the ANOVA test, Figure 3). Additionally, the protein expression of Bax was the same in the AZA only group and the control group (p>0.1 by the ANOVA test, Figure 3). The ratio of Bcl2/Bax was higher in the AZA+CSE group than in the CSE group (1.95 ± 0.07 vs 0.33 ± 008; p<0.01 by the ANOVA test). These results suggest that the regulation of Bax expression might be controlled by a mechanism other than gene methylation.

### Inhibiting DNA methylation alleviates CSE– induced endothelial apoptosis

**Figure 4:**
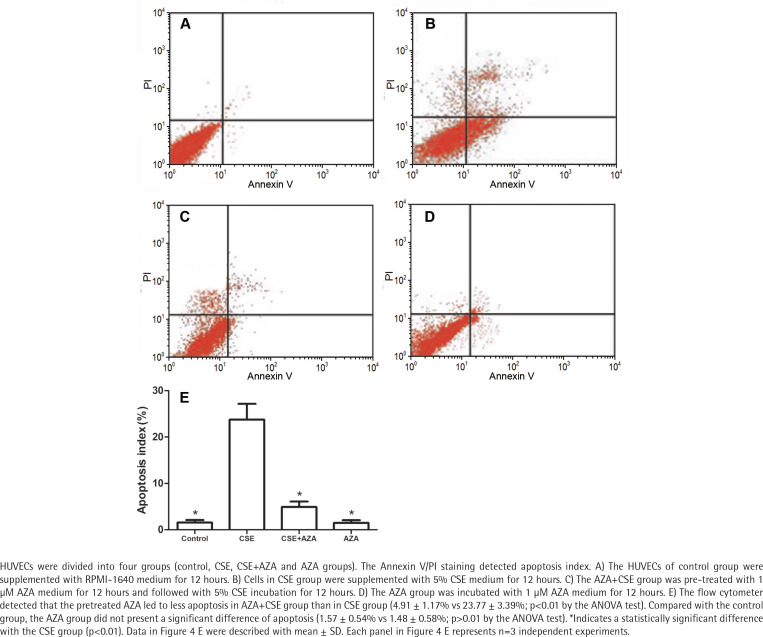
Inhibiting of DNA methylation alleviates CSE-induced endothelial apoptosis

The flow cytometer detected that the cells in the AZA + CSE group had a lower degree of apoptosis than the cells in the CSE group (p<0.01 by the ANOVA test, Figure 4). Apoptosis in the AZA group was not significantly different than that in the control group (p>0.1 by the ANOVA test, Figure 4). It seems that the single use of AZA may not cause more apoptosis than the control treatment. Notably, the abovementioned results demonstrate that inhibiting DNA methylation treatment alleviates CSE-induced endothelial apoptosis.

## DISCUSSION

Cigarette smoke is inhaled into the lungs and diffuses into the alveoli and blood. The first barrier is the mucus on the surface of the airways and alveoli. The cigarette smoke inhaled into lungs diffuses and dissolves into mucus. The mucus is a kind of liquid, and CSE could mimic this liquid phase better than cigarette smoke. Notably, cigarette smoke is a risk factor for many diseases, such as coronary artery disease, hypertension, rheumatoid arthritis and chronic obstructive pulmonary disease (COPD), among others^20,22,23^. Our group also found injury of the cardiac and respiratory system in CSE-induced emphysema models^1^. All of the abovementioned studies indicate that cigarette smoke could induce multisystem damage, not only to the pulmonary system. The endothelia cells might be a direct target for cigarettes, and HUVECs are probably the most accepted models for human endothelial cells^24-26^ and are widely used in endothelial research^27,28^. This study found that CSE induced the apoptosis of HUVECs, which is consistent with results of previous studies^4,13,14,29^. Furthermore, this study found Bcl-2 hypermethylation after CSE incubation, and inhibiting methylation could reverse the apoptosis and hypermethylation. Prior studies have documented that endothelial apoptosis plays a role in different diseases of different systems^30-33^. It is possible that cigarette smoke might cause multisystem diseases, or at least endothelium-associated diseases, through endothelial apoptosis.

Mechanistically, it is widely accepted that Bcl-2 family proteins regulate apoptosis through the release of cyt C from the mitochondria. Bcl-2, a principal anti-apoptotic protein, is an agonist of the pro-apoptotic protein Bax. It has been demonstrated that decreased Bcl-2/Bax causes the release of cytosolic cyt C from mitochondria, eventually triggering apoptosis^34^. As in previous studies^35,36^, our group found CSE treatment decreased the expression of Bcl-2 and the Bcl-2/Bax ratio and increased cytosolic cyt C protein levels. The imbalance between Bcl-2 and Bax coincided with increased endothelial apoptosis. These results suggest that CSE might induce endothelial apoptosis by reducing Bcl-2 expression, resulting in the release of cytosolic cyt C.

We found that the downregulation of Bcl-2 protein levels was consistent with mRNA levels and gene methylation status. The results also showed that CSE incubation led to higher methylation of the Bcl-2 promoter. Furthermore, inhibiting DNA methylation restored CSE-induced hypermethylation of the Bcl-2 promoter and mRNA expression. CpG methylation, in which methyl groups are attached to cytosine bases next to guanine (CpG site), is an emerging and important pre-transcriptional regulation mechanism^37^. Promoter methylation inhibits transcription factor binding and target gene transcription, which blocks gene expression. According to the Hoffman and Hu^21^ research of the CpG island, this work detected an important promoter element of human Bcl-2 located within P2. Hypermethylated P2 will turn off and silence Bcl-2 expression^21^. Our results suggest that CSE might lower Bcl-2 expression by increasing the methylation status of the Bcl-2 promoter.

In addition, we demonstrated that the AZA alleviated CSE-induced endothelial apoptosis by restoring Bcl-2 methylation status. It might be deduced that CSE-induced hypermethylation of the Bcl-2 promoter participates in endothelial apoptosis, even in endothelium-associated diseases. Since DNA methylation patterns are mostly not heritable, they can result from many factors, such as the environment, food, and stress. The DNA methylation status can be different in populations with the same genetic background. The abovementioned deduction can explain why only certain smokers suffer cigarette-induced diseases within genetically related populations, while others with the same genetic background do not develop diseases. Once DNA methylation develops, it will stably propagate during a lifetime, leading to a persistent downregulation of gene expression. The propagation of DNA methylation can also explain why some cigarette-induced diseases, for example COPD, continue progressing after smoke cessation. However, AZA treatment demethylates many genes, not just Bcl-2. To clarify whether the hypermethylation of the Bcl-2 promoter directly induces endothelial apoptosis, additional studies to specifically target the methylation and demethylation of the Bcl-2 promoter are required. The CRISPR system precisely and efficiently mutates genes through sgRNA and Cas^38^. Exogenous plasmids can also mutate genes in precise loci. Both gene editing methods can identify the exact hypermethylated loci and demonstrate the effect of promoter methylation. In a future study, the CRISPR system might be an appropriate method to clarify the function of Bcl-2 methylation.

The protein levels of Bax, the proapoptotic member of the Bcl-2 family, were increased after CSE incubation in this study. AZA did not reverse the pattern of Bax expression, and Bax mRNA levels showed no difference between the CSE and control groups. The results suggest that the CSE-induced high expression of Bax might be regulated after transcription or even translation, but further study is required.

## CONCLUSIONS

This study presents the novel result that inhibiting DNA methylation can alleviate CSE-induced endothelial apoptosis and Bcl-2 promoter methylation. Furthermore, Bcl-2 promoter methylation might be involved in CSE-induced endothelial apoptosis.
