# Supplementary material for: Inhibiting DNA methylation alleviates cigarette smoke extract-induced dysregulation of Bcl-2 and endothelial apoptosis
Source: Tob Induc Dis. 2020 Jun 3;18:51. doi: 10.18332/tid/119163 (PMC7291961; doi:10.18332/tid/119163)
Supplement: Supplementary file 1 [file TID-18-51-s1.pdf]

1. CSE concentration pilot study

After starvation, PBS, 2.5% CSE, 5% CSE, 10% and 20%CSE were used to incubate the HUVECs 12 hours. The result showed that The CSE induced HUCECs apoptosis was concentration dependent. However, the cell necrosis also depended on concentration. 5% CSE induced the higher apoptosis cells than 2.5% CSE (25.73±5.07% vs. 11.50±2.01%), and less dead or necrosis cells than 10% CSE (7.2±2.54% vs. 13.9±9.78%) (S. table 1). Furthermore, 5% CSE did not lead significant more dead or necrosis cells then 2.5% CSE. Because of the above pilot study results, we choose the 5% CSE to treat cells.

Supplement Table 1 Apoptosis and necrosis in different CSE concentration group.

CSE concentration	Apoptosis cells (%)	Dead or Necrosis cells (%)
PBS	1.57±0.54	0.97±0.47
2.5%	11.50±2.0251*	5.93±1.74*
5.0%	25.73±5.07*#	7.2±2.54*
10.0%	46.13±5.07*#	13.9±9.78*#
20.0%	21.60±1.55*#	45±12.91*#

* indicates a statistically significant difference compared with PBS group (P<0.01 by the ANOVA test). # indicates a statistically significant difference compared with 2.5% CSE group (P<0.01 by the ANOVA test).

© 2020 Zeng H. et al.
